# Gas film retention and underwater photosynthesis during field submergence of four contrasting rice genotypes

**DOI:** 10.1093/jxb/eru166

**Published:** 2014-04-23

**Authors:** Anders Winkel, Ole Pedersen, Evangelina Ella, Abdelbagi M. Ismail, Timothy D. Colmer

**Affiliations:** ^1^School of Plant Biology, The University of Western Australia, 35 Stirling Highway, Crawley, WA 6009, Australia; ^2^Institute of Advanced Studies, The University of Western Australia, 35 Stirling Highway, Crawley, WA 6009, Australia; ^3^Freshwater Biological Laboratory, Department of Biology, University of Copenhagen, Universitetsparken 4, 3rd floor, 2100 Copenhagen, Denmark; ^4^International Rice Research Institute, DAPO Box 7777, Metro Manila, the Philippines

**Keywords:** Aerenchyma, flooding stress, leaf gas films, leaf air layer, leaf hydrophobicity, *Oryza sativa*, submergence tolerance, *SUB1*, leaf chlorophyll, survival, FR13A, IR42, Swarna, Swarna-Sub1.

## Abstract

Floods can completely submerge some rice (*Oryza sativa* L.) fields. Leaves of rice have gas films that aid O_2_ and CO_2_ exchange under water. The present study explored the relationship between gas film persistence and underwater net photosynthesis (P_N_) as influenced by genotype and submergence duration. Four contrasting genotypes (FR13A, IR42, Swarna, and Swarna-Sub1) were submerged for 13 days in the field and leaf gas films, chlorophyll, and the capacity for underwater P_N_ at near ambient and high CO_2_ were assessed with time of submergence. At high CO_2_ during the P_N_ assay, all genotypes initially showed high rates of underwater P_N_, and this rate was not affected by time of submergence in FR13A. This superior photosynthetic performance of FR13A was not evident in Swarna-Sub1 (carrying the *SUB1* QTL) and the declines in underwater P_N_ in both Swarna-Sub1 and Swarna were equal to that in IR42. At near ambient CO_2_ concentration, underwater P_N_ declined in all four genotypes and this corresponded with loss of leaf gas films with time of submergence. FR13A retained leaf gas films moderately longer than the other genotypes, but gas film retention was not linked to *SUB1*. Diverse rice germplasm should be screened for gas film persistence during submergence, as this trait could potentially increase carbohydrate status and internal aeration owing to increased underwater P_N_, which contributes to submergence tolerance in rice.

## Summary

The flood-tolerant genotype FR13A retains leaf gas films and its capacity for underwater net photosynthesis, whereas gas films are lost faster and photosynthesis declines markedly in sensitive genotypes.

## Introduction

Flooding severely impedes gas exchange between plants and the environment owing to the 10^4^-fold slower diffusion of gases in water compared with in air (Armstrong, 1979). Rain-fed lowland rice is a semi-aquatic plant that often becomes submerged, but genotypes differ markedly in tolerance (Colmer *et al.*, 2014; Ram *et al.*, 1999). FR13A is a submergence-tolerant landrace and much of this tolerance is conferred by a major QTL (quantitative trait locus) called ‘*SUB1*’ (Xu and Mackill, 1996). The *SUB1* QTL controls several traits contributing to submergence tolerance, including reduced shoot elongation, maintenance of higher soluble carbohydrate concentration, and less chlorophyll degradation during submergence, as well as less oxidative stress post-submergence (Ella *et al.*, 2003a; Ella *et al.*, 2003b). Rice genotypes with *SUB1* therefore show better survival and recovery post-submergence than those lacking this QTL (Bailey-Serres *et al.*, 2010; Ismail *et al.*, 2013; Mackill *et al.*, 2012). SUB1A-1 is an ERF transcriptional regulator that blocks ethylene responsiveness during submergence and thus also down-stream targets. It maintains the expression of the gibberellic acid (GA) signalling repressors SLENDER RICE1 (*SLR1*) and SLR1-like-1 (*SLRL1*) and their proteins during submergence. Expression of these repressors is associated with inhibition of GA induction of expansins required for cell wall expansion, and α-amylase and sucrose synthase required for starch and sucrose catabolism, respectively (Bailey-Serres *et al.*, 2010; Fukao and Bailey-Serres, 2008; Fukao *et al.*, 2006). More recently, Schmitz *et al.* (2013) reported that SUB1 differentially regulates genes associated with brassinosteroids (BR) synthesis, and BR induces a GA catabolic gene, *GA2ox7*, under submergence. Together these processes lead to suppression of GA-induced underwater elongation growth and conserve carbohydrates for maintenance metabolism and survival.

In addition to the importance placed on conserving carbohydrates during submergence (Bailey-Serres and Voesenek, 2008; Voesenek *et al.*, 2006), many wetland plants can also produce carbohydrates through underwater photosynthesis (Colmer *et al.*, 2011; Mommer *et al.*, 2004). Rice, in particular, has been shown to photosynthesize under water (Raskin and Kende, 1983; Setter *et al.*, 1989) and rice grew well when submerged in water enriched with CO_2_ to levels above air equilibrium to simulate some floodwaters (Pedersen *et al.*, 2009; Setter *et al.*, 1989). Like several other terrestrial wetland plants (Colmer and Pedersen, 2008b), rice possesses superhydrophobic, self-cleansing leaf surfaces that retain a thin gas film when immersed into water (Pedersen *et al.*, 2009; Raskin and Kende, 1983; Setter *et al.*, 1989). Leaf gas films markedly enhance gas exchange between leaf and floodwater so that underwater net photosynthesis (P_N_) is greater for leaves with gas films present, than when these are removed (Pedersen *et al.*, 2009; Verboven *et al.*, 2014; Winkel *et al.*, 2013). In addition to carbohydrate production, underwater P_N_ also results in better root aeration as much of the O_2_ produced in the leaves diffuses via the aerenchyma down to the roots (Colmer and Pedersen, 2008a; Pedersen *et al.*, 2009; Waters *et al.*, 1989; Winkel *et al.*, 2013). As O_2_ production in underwater P_N_ ceases at dusk, leaf gas films then also facilitate O_2_ uptake from the floodwater resulting in some internal aeration during darkness, but this is likely to be insufficient for the entire root system as root O_2_ decreases to very low levels and fermentation occurs during dark periods (Pedersen *et al.*, 2009; Waters *et al.*, 1989; Winkel *et al.*, 2013).

*SUB1* genotypes show less chlorophyll degradation during submergence (Ella *et al.*, 2003b), but the possible benefit of this to underwater P_N_ has not previously been evaluated. Furthermore, whether the leaves of submergence-tolerant FR13A or *SUB1* lines differ from sensitive rice genotypes in formation and/or maintenance of leaf gas films should be evaluated. The issue of underwater P_N_ in FR13A and *SUB1* genotypes is important to evaluate as the *SUB1* QTL accounts for 70% of the variation in submergence tolerance leaving 30% unexplained variation (Xu and Mackill, 1996). We assessed the submergence tolerance of 4 selected genotypes of rice during 13 d of complete submergence. The four genotypes were (i) FR13A (the tolerant donor of *SUB1A*), (ii) IR42 (submergence intolerant and lacking *SUB1A*), (iii) Swarna (submergence intolerant and lacking *SUB1A*), and (iv) Swarna-Sub1 (Swarna with *SUB1A*). Over the period of 13 d of complete submergence in an experimental field, we followed with time underwater P_N_, leaf chlorophyll concentrations, and leaf gas film thickness for the four contrasting genotypes in order to elucidate: (a) relationships between loss of chlorophyll and/or gas film persistence with underwater P_N_ capacity (i.e. at near-saturated CO_2_) and at near-ambient CO_2_ (i.e. field-relevant), as influenced by time of submergence; and (b) if FR13A is superior in its capacity for underwater P_N_ whether this trait is also expressed in Swarna-Sub1.

## Materials and methods

### Experimental design and harvest procedures

The submergence experiment was conducted in the wet season (Oct to Nov) in the submergence field facilities at the International Rice Research Institute at Los Baños, the Philippines, with field and soil type described previously (Singh *et al.*, 2009). Rice genotypes (*Oryza sativa* L.; FR13A, IR42, Swarna and Swarna-Sub1) were sown in a seedbed in September 2011 and 21-d-old seedlings were transplanted at 20×20cm spacing into a waterlogged paddy field surrounded by bunds to enable submergence to be imposed. FR13A is a landrace from eastern India with exceptional submergence tolerance and is the donor of *SUB1*, a major QTL associated with submergence tolerance on chromosome 9; IR42 is a submergence-intolerant variety (Mackill *et al.*, 2012). Swarna is a dwarf rain-fed lowland Indian variety and Swarna-Sub1 is Swarna with the *SUB1* QTL introgressed through marker assisted backcrossing for improvement of submergence tolerance (Xu *et al.*, 2006). Experiments commenced 14 d after transplanting, so that plants were 5 weeks old. Plants were completely submerged with about 1.25 m of water head and remained inundated through to the end of the experiment.

Plants were sampled at various times after submergence (see Figures) for analyses of underwater net photosynthesis (P_N_), leaf (lamina) chlorophyll concentrations, and lamina gas film thickness. Measurements were also taken of lamina sugar and starch concentrations, tissue porosity, and of whole shoot dry mass (DM); these supporting data are in the Supplementary Materials. A floating air-filled mattress was used to access plants in the submergence pond as this avoided disturbance of the soil that would have resulted in suspended particles and murky water; plants were gently pulled out of the soil and immediately submerged in floodwater from the same field in a plastic container to prevent air contact. This procedure did not capture all root material and thus roots were not included in any tissue analyses. Immediately after collection, plants were brought to the laboratory for analyses.

### Environmental conditions

Water used to submerge the paddy field came from an adjacent reservoir; see Winkel *et al.* (2013) for key water chemical parameters. Morning water temperature in the paddy field was measured between 9.00h and 10.00h each day and ranged from 28–30 °C; the average O_2_ concentration (for the 12 mornings) was 195 mmol m^–3^ (17 kPa); air-equilibrium at 30 °C is 254 mmol m^–3^ or 20.6 kPa. Average alkalinity in the water was 5.4mol m^–3^ and pH was 7.9, resulting in an average dissolved CO_2_ concentration of 130 mmol m^–3^ for the 12 mornings of the experiment. The CO_2_ concentration in the study of Winkel *et al.* (2013) declined, relative to the morning value, to 71% by midday and then further to 53% by dusk. Light extinction in the water ranged from 1.1–1.9 m^–1^ with an average of 48% of surface light remaining at 50cm of depth (depth of floodwater was approximately 1.25 m, average initial plant height varied from 37 to 77cm). During the 13 d of submergence, the average air temperature was 26.7 °C, and varied from 23.3–32.7 °C. Average incident radiation was 403W m^–2^ in the period from 10.00 h–14.00h for the 13 days of submergence.

### Net photosynthesis under water and in air

Underwater P_N_ was measured on excised leaf (lamina) segments at 0.2 and 5mol m^–3^ of CO_2_. These two CO_2_ concentrations were chosen based on: (i) 0.2mol m^–3^ represents a reasonable near-ambient CO_2_ concentration in rice floodwaters—these waters typically contain CO_2_ above air-equilibrium concentrations during early mornings owing to night-time CO_2_ production, although CO_2_ can be depleted below air-equilibrium by the afternoon (summarized in Colmer *et al.* (2011), dynamics in Winkel *et al.* (2013)); (ii) five mol m^–3^ CO_2_ saturates underwater P_N_ of rice, irrespective of leaf gas films presence or absence (Swarna-Sub1; Winkel *et al.*, 2013) and so these measurements enabled the evaluation of the maximum capacity for underwater P_N_ in the present system, and how this changed with time. Although 5mol m^–3^ CO_2_ would be regarded as a very high level of CO_2_ (possibly with some adverse effects on cellular metabolism) if in a gas phase (viz. 5mol m^–3^ is equivalent to 17.2 kPa CO_2_ in equilibrium with air at 30 °C), the CO_2_-response curve for underwater P_N_ did not show any adverse effects of this high CO_2_ (Winkel *et al.*, 2013). The resistance of transversing an aqueous diffusive boundary layer (DBL) is 10 000 times that of an equivalent gaseous DBL and so the CO_2_ concentration experienced by the cells of photosynthesizing leaves (consuming CO_2_) would be substantially lower when submerged than if in a gas phase of equivalent CO_2_.

Four replicate leaves (the second youngest fully expanded from four different plants) were taken from each of the four genotypes. Twenty mm-long leaf segments (projected area of approximately 200mm^2^) were excised from the top third of the lamina. Underwater P_N_ (*n*=4) was measured at 30 °C using 25ml glass vials with two glass beads added to ensure mixing according to the method of Pedersen *et al.* (2013) with PAR inside the vials of 760±60 µmol m^–2^ s^–1^ (mean±SE, *n=*10). The incubation medium was artificial floodwater based on a general purpose culture medium of Smart and Barko (1985) modified by Colmer and Pedersen (2008a), with initial O_2_ near half air-equilibrium. To prepare artificial floodwater with a final concentration of 0.2 or 5mol m^–3^ CO_2_ and an alkalinity of 5mol m^–3^ (mostly bicarbonate and carbonate), we added KHCO_3_ at 5.2 or 10.0mol m^–3^ in the general purpose medium. We subsequently added known volumes of 0.1M HCl to convert the desired portion of the HCO_3_^–^ into CO_2_, resulting in pH values of 7.7 and 6.3 for the 0.2 and 5mol CO_2_ m^–3^, respectively (Mackereth *et al.*, 1978). Vials without leaf segments served as blanks.

Following incubations of known durations (30–50min), the dissolved O_2_ concentration in each vial was measured using an O_2_ minielectrode (OX-500, Unisense A/S, Aarhus, Denmark) connected to a multimeter (MicroSensor Multimeter, Unisense A/S, Aarhus, Denmark). Fresh mass (FM) was then taken before samples were flash frozen in liquid N_2_ and freeze-dried and DM recorded. A relationship between DM and area, and also for FM and area, was established for segments from the same type of leaves for each individual genotype, for plants when waterlogged with leaves in air and also when submerged, using digital photos and ImageJ (Schneider *et al.*, 2012), so that the projected area of each leaf segment used in underwater P_N_ could be calculated from its DM. Using the differences between DM to area ratio from the field plants in waterlogged soil with shoots in air or when completely submerged, a linear correction was calculated to estimate the change in DM to area ratio during the submergence.

P_N_ in air by plants in waterlogged soil with shoots that always remained in air was measured on each of the four genotypes on the second youngest fully expanded leaf using an IRGA (LI-6400, Li-Cor) at PAR of 750 µmol m^–2^ s^–1^ and CO_2_ (380 µL L^–1^) at 30 °C between 10 and 11 am in the adjacent waterlogged paddy field; for details see Winkel *et al.* (2013).

### Gas film thickness

Gas film volume was measured by determining buoyancy of lamina samples before and after gas film removal. Measurements were taken on three segments of 50mm length of the lamina from the top third of the youngest fully expanded leaf of 3 tillers. After the first measurement of buoyancy (gas films intact) segments were brushed with a dilute solution of Triton X (0.01% v/v of Triton X-100 in artificial floodwater, composition given above) to eliminate hydrophobicity so that gas films were removed (c.f. Colmer and Pedersen, 2008b; Pedersen *et al.*, 2009) and thereafter buoyancy was again measured. The samples were then vacuum infiltrated with water and again measured for buoyancy, to enable calculation of tissue porosity (gas-filled volume per unit tissue volume; Raskin, 1983). Segment area was calculated from the area to FM ratio, which was established for similar tissues (described above). Mean gas film thickness was calculated by dividing gas film volume (mm^3^) with the two-sided area (mm^2^), i.e. rice leaves possess gas films on both the adaxial and abaxial sides (Pedersen *et al.*, 2009). In the present study, the detection limit of gas film thickness was approximately 2 µm and so measurements giving values below 2 µm were classified as “gas films absent”.

### Chlorophyll

Chlorophyll concentration was measured on the middle portion of the 2nd youngest fully expanded leaf of individual plants harvested from the submerged field. The samples were flash frozen in liquid N_2_, freeze-dried for 48h, stored at –80 °C and then ground. Chlorophyll was extracted in 80% acetone at 5 °C for 12h in darkness and then absorbance in extracts was measured at 645, 652, and 663nm on a spectrophotometer (UV-VIS 1800, Shimadzu, Nishinokyo, Kyoto, Japan). Chlorophyll concentrations were calculated using equations of Mackinney (1941).

### Statistical analyses

GraphPad Prism 6 (GraphPad Software Inc., http://www.graphpad.com) was used for data analysis and two-way ANOVA with Bonferroni *post hoc* test to compare means of the differences in sugar, starch (in Supplementary Data, only), underwater P_N_, gas film thickness, and chlorophyll of the leaves of the four genotypes. Analyses of two-way ANOVA were performed separately for FR13A versus IR42 and Swarna versus Swarna-Sub1 to enable better interpretation of potential factorial interactions. Correlations between underwater P_N_ at the two CO_2_ concentrations and gas film thickness and tissue chlorophyll concentration were also performed using GraphPad Prism 6 (Spearman non-parametric correlation).

## Results

### Capacity for underwater net photosynthesis; measurements at high dissolved CO_2_ (5mol m^–3^)

**Fig. 1.**
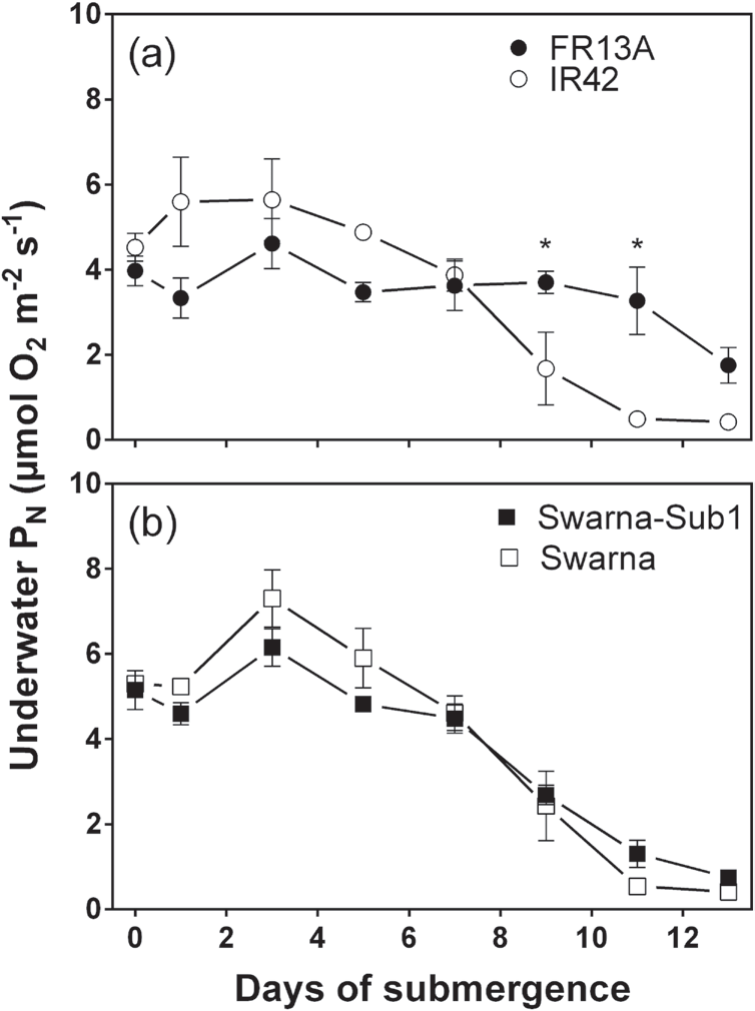
Underwater net photosynthesis (P_N_) of four genotypes of 5–7 weeks old rice (*Oryza sativa*) with time of submergence. (a) FR13A (submergence tolerant and donor of *SUB1*) and IR42 (submergence intolerant) and (b) Swarna (submergence intolerant) and Swarna-Sub1 (submergence tolerant with SUB1 QTL introgressed). Lamina segments of 蝤200mm^2^ were incubated in rotating glass vials with 5mol CO_2_ m^–3^ and PAR of 760 µmol photons m^–2^ s^–1^ at 30 °C and P_N_ was measured as O_2_ evolution (mean±SE, *n*=4). Underwater P_N_ decreased significantly with time of submergence (Table 1); asterisk denotes significant differences between the two genotypes in each panel (Bonferroni test). Photosynthetic rates in air by FR13A, IR42, Swarna-Sub1 and Swarna, were 32.9±2, 40.3±3.4, 33.8±2.3, and 37.0±1.3 µmol CO_2_ m^–2^ s^–1^, and were not significantly different (1-way ANOVA, means±SE, *n*=3–9).

Measurements of underwater P_N_ with 5mol CO_2_ m^–3^, a level that saturates underwater P_N_ of Swarna-Sub1 (irrespective of leaf gas films presence or absence) in the present system (Winkel *et al.*, 2013), was used to evaluate changes in capacity for underwater P_N_ with time after submergence. All four genotypes had initial maximal underwater P_N_ values between 4.0 and 5.3 µmol O_2_ m^–2^ s^–1^ (no significant difference; Fig. 1a, b). Capacity for underwater P_N_ by FR13A and IR42 was significantly affected by time of submergence but maximal underwater P_N_ of IR42 declined faster during the second week of submergence so that by the 13th day the rate was only 9% of the initial capacity (Fig. 1a; Table 1). Thus, during the latter part of the submergence treatment, capacity for underwater P_N_ by FR13A was 6.7-fold higher than in IR42 (Fig. 1a). This superior performance of FR13A for retention of underwater photosynthetic capacity was not evident in Swarna-Sub1, which contains the SUB1 QTL from FR13A (Fig. 1b). The declines in capacity for underwater P_N_ with time of submergence, in both Swarna-Sub1 and Swarna were equal to that in IR42 (Fig. 1a, b and Table 1). With high external CO_2_ in the floodwater, P_N_ under water was 13.4–19.5% of ambient rates in air (rates of P_N_ in air are given in the caption of Fig. 1). The lower P_N_ rates under water than in air probably results from a combination of high resistance to gas exchange even in the presence of leaf gas films (Verboven *et al.*, 2014) impeding O_2_ exit that is further reduced by the relatively low solubility of O_2_ in water, which would result in O_2_ build-up inside the tissues, and thus high photorespiration under water, as previously discussed for rice by Setter *et al.* (1989).

**Table 1.** Key-results of 2-way ANOVA tests related to data shown in Figures 1, 2, 4, and 6. Analyses were performed for each parameter studied (underwater P_N_ at 5 and 0.2mol CO_2_ m^–3^, gas film persistence, and leaf chlorophyll) with two genotypes (FR13A versus IR42 or Swarna versus Swarna-Sub1). *P*- and *F*-values are given for “genotype”, “time” and “genotype × time”. A *P*-level of 0.05 was used, but *P*-values for *P*<0.1 are also shown in italics; n.s.=not significant. Abbreviations: UW=underwater; P_N_=net photosynthesis; Chl=total chlorophyll.

Parameters and genotype pairs in comparisons	“genotype”		“time”		“genotype × time”		Data in Figure number
	*P*-value	*F*-value	*P*-value	*F*-value	*P*-value	*F*-value	
UW P_N_ 5 FR13A vs. IR42	n.s.	0.1	<0.0001	11.7	0.0003	5.0	1
UW P_N_ 5 Swarna vs. Swarna-Sub1	n.s.	1.2	<0.0001	60.6	n.s.	1.4	1
Chl FR13A vs. IR42	0.0009	12.1	<0.0001	52.9	<0.0001	17.4	2
Chl Swarna vs. Swarna-Sub1	0.030	4.9	<0.0001	69.9	0.0003	4.3	2
UW P_N_ 0.2 FR13A vs. IR42	0.058	8.3	<0.0001	46.1	<0.0001	5.7	4
UW P_N_ 0.2 Swarna vs. Swarna-Sub1	n.s.	0.8	<0.0001	45.3	n.s.	0.4	4
Gas film FR13A vs. IR42	0.071	3.9	<0.0001	50.5	<0.0001	5.9	6
Gas film Swarna vs. Swarna-Sub1	0.069	3.4	<0.0001	62.3	0.052	2.0	6

**Fig. 2.**
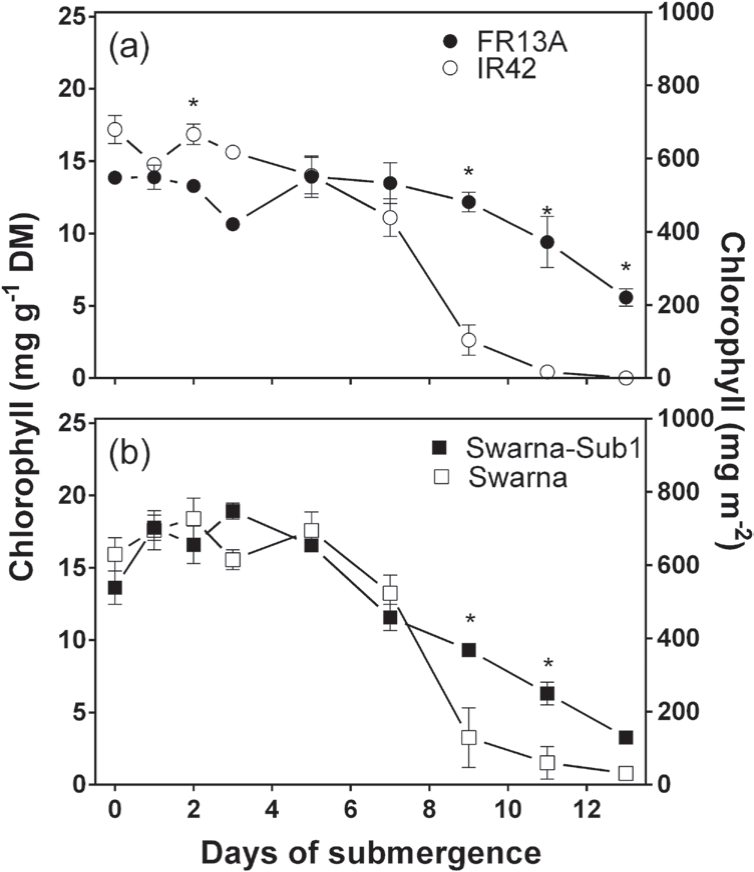
Total chlorophyll concentration of four genotypes of 5–7 weeks old rice (*Oryza sativa*) with time of submergence. (a) FR13A (submergence tolerant and donor of *SUB1*) and IR42 (submergence intolerant) and (b) Swarna (submergence intolerant) and Swarna-Sub1 (submergence tolerant with *SUB1* QTL introgressed). Chlorophyll concentration was measured on the middle portion of the 2nd youngest fully expanded leaf (mean±SE, *n*=4). Chlorophyll concentration decreased significantly with time of submergence for all four genotypes (Table 1); asterisk denotes significant differences between the two genotypes in each panel (Bonferroni test).

Declines in leaf chlorophyll concentrations with time of submergence (Fig. 2a, b), as well as other possible changes in the photosynthetic apparatus (not studied here), presumably contributed to the decline in photosynthetic capacity (Fig. 1a, b). Genotypes did not differ significantly in initial chlorophyll concentration. In all four genotypes, leaf chlorophyll declined with time of submergence but the patterns of these declines differed (Fig. 2a, b). Similar with the pattern for underwater photosynthetic capacity, FR13A and IR42 did not differ in chlorophyll concentrations during the first 8 days of submergence, but later in the submergence period the values in IR42 fell well below those in FR13A (Fig. 2a and Table 1). Interestingly, the superior chlorophyll retention of FR13A was conferred by the *SUB1* QTL when in the Swarna background (Fig. 2b; i.e. Swarna-Sub1). The decline in leaf chlorophyll with time of submergence in Swarna did not differ from that in IR42 (Fig. 2a, b), whereas in Swarna-Sub1 it was more similar to FR13A.

**Fig. 3.**
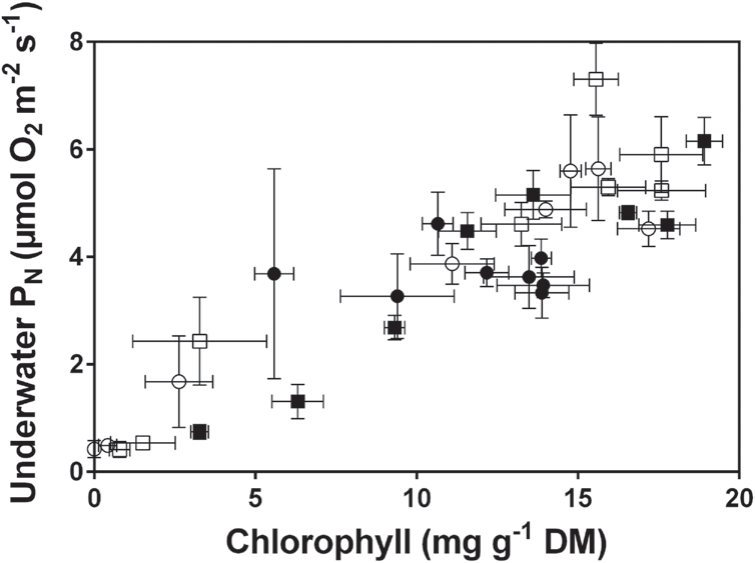
Total chlorophyll concentration versus underwater net photosynthesis (P_N_) measured at 5mol CO_2_ m^–3^ of four genotypes of 5–7 weeks old rice (*Oryza sativa*). Genotypes were: FR13A (submergence tolerant and donor of *SUB1*; solid circle), IR42 (submergence intolerant; open circle), Swarna (submergence intolerant; open square), and Swarna-Sub1 (submergence tolerant with *SUB1* QTL introgressed; solid square). Spearman rank correlation analyses (one-tailed) of chlorophyll concentration versus underwater P_N_ showed: all genotypes pooled, *P*<0.0001; FR13A *P*=0.3517; IR42 *P*=0.0054; Swarna *P*=0.0140, and Swarna-Sub1 *P*=0.0023. Means±SE, *n*=5.

Correlation analyses were used to evaluate the relationships between leaf chlorophyll concentrations and capacity for underwater P_N_ (Fig. 3). Underwater P_N_ was positively correlated with leaf chlorophyll concentration for IR42, Swarna-Sub1, and Swarna, but not for FR13A. FR13A, in contrast with the other three genotypes, did not show a decline in underwater P_N_ (Fig. 1a) despite that leaf chlorophyll decreased to 68% of its initial concentration on day 11 and to 40% on day 13 (Fig. 2a). If the submergence period was extended, so FR13A suffered greater declines in chlorophyll similar to those already apparent in the other three genotypes, then underwater P_N_ would presumably decline and also result in a positive correlation between chlorophyll and underwater P_N_ in FR13A.

Although changes in leaf chlorophyll concentration, and possibly other changes in the photosynthetic machinery, presumably were the major factors contributing to declines in capacity for underwater P_N_ (Fig. 3), it should also be noted that towards the end of the submergence period (day 10 onwards), the previously gas-filled volume of the tissue had been infiltrated by water in three of the four genotypes (Supplementary Fig. S1 available at *JXB* online), the exception was FR13A. Water infiltration of the leaf tissue is an indication of structural degradation; any such tissue degradation would also have contributed to the low chlorophyll concentrations (Fig. 2) and very low rates of underwater P_N_ (even at 5mol CO_2_ m^–3^) of IR42, Swarna, and Swarna-Sub 1 at the end of the treatment period (Fig. 1a, b).

### Underwater net photosynthetic rates at near-ambient dissolved CO_2_ (0.2mol m^–3^)

Measurements of underwater P_N_ with 0.2mol CO_2_ m^–3^, a near ambient concentration in a similar field situation (Winkel *et al.*, 2013), was used to evaluate field relevant rates of underwater P_N_ with time after submergence. At this CO_2_ concentration, underwater P_N_ is limited by CO_2_ entry owing to the high resistance to diffusion from the bulk medium into the submerged leaf (Pedersen *et al.*, 2009; Winkel *et al.*, 2013). Therefore, gas film presence, a feature which reduces gas exchange resistance of submerged leaves (Colmer and Pedersen, 2008b; Raskin and Kende, 1983; Verboven *et al.*, 2014), is of importance. Thus, the relationship of gas film persistence with underwater P_N_, and decline in leaf chlorophyll concentrations, both as influenced by time of submergence, are of importance to characterize for contrasting genotypes. To facilitate comparison with non-limiting CO_2_ conditions, we first consider the photosynthetic rates at near-ambient dissolved CO_2_ as related to the decline in leaf chlorophyll (Fig. 2a, b) and then followed by consideration of the role of leaf gas films.

**Fig. 4.**
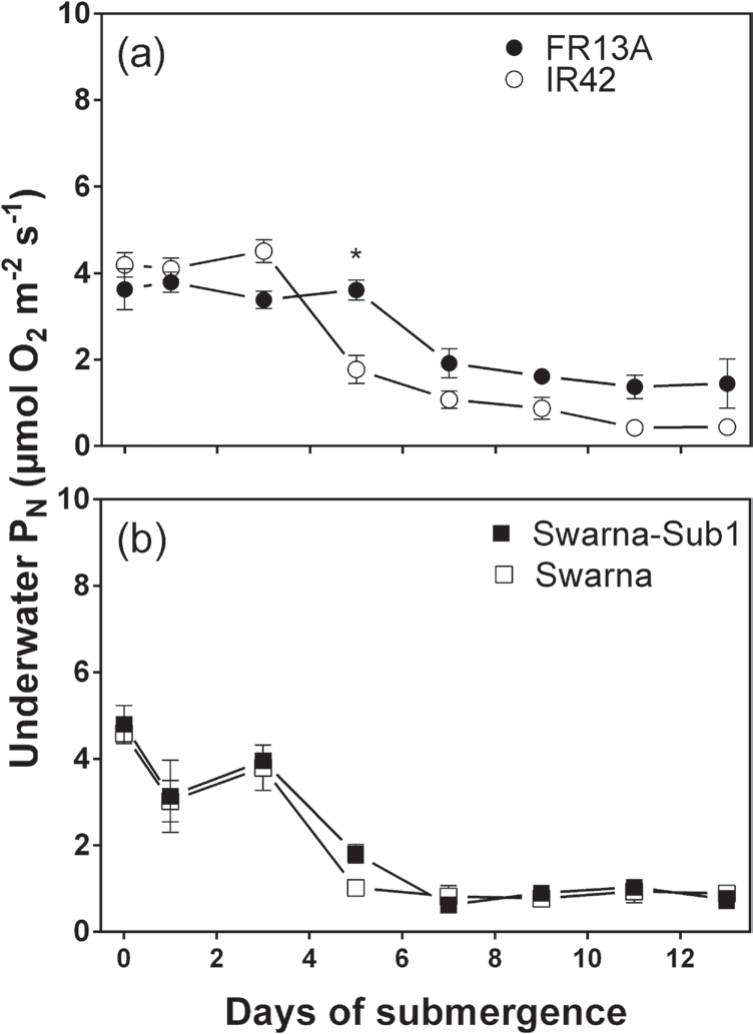
Underwater net photosynthesis (P_N_) of four genotypes of 5–7 weeks old rice (*Oryza sativa*) with time of submergence. (a) FR13A (submergence tolerant and donor of *SUB1*) and IR42 (submergence intolerant) and (b) Swarna (submergence intolerant) and Swarna-Sub1 (submergence tolerant with *SUB1* QTL introgressed). Lamina segments of 蝤 200mm^2^ were incubated in rotating glass vials with 0.2mol CO_2_ m^–3^ and PAR of 760 µmol photons m^–2^ s^–1^ at 30 °C and P_N_ was measured as O_2_ evolution (mean±SE, *n*=4). Underwater P_N_ decreased significantly with time of submergence for all four genotypes (Table 1); asterisk denotes significant differences between the two genotypes in each panel (Bonferroni test).

**Fig. 5.**
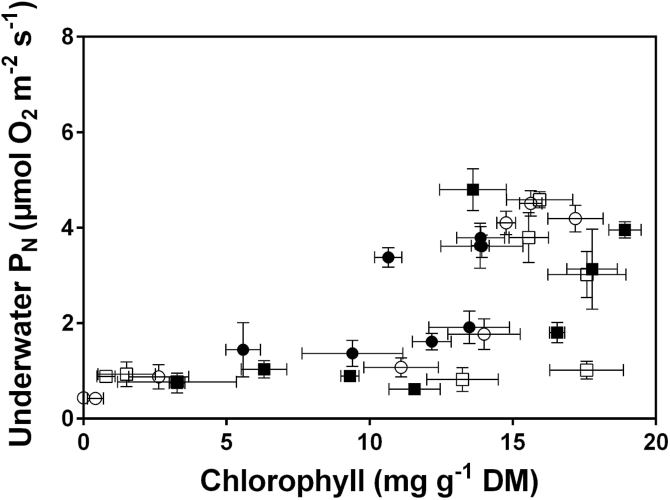
Total chlorophyll concentration versus underwater net photosynthesis (P_N_) measured at 0.2mol CO_2_ m^–3^ of four genotypes of 5–7 weeks old rice (*Oryza sativa*). Genotypes were: FR13A (submergence tolerant and donor of *SUB1*; solid circle), IR42 (submergence intolerant; open circle), Swarna (submergence intolerant; open square), and Swarna-Sub1 (submergence tolerant with *SUB1* QTL introgressed; solid square). Spearman rank correlation analyses (one-tailed) of chlorophyll concentration *versus* underwater P_N_ showed: all genotypes pooled, *P*<0.0001; FR13A *P*=0.0077; IR42 *P*=0.0006; Swarna *P*=0.0575, and Swarna-Sub1 *P*=0.0347. Means±SE, *n*=5.

All four genotypes had initial underwater P_N_ rates of 3.6–4.8 µmol O_2_ m^–2^ s^–1^ (no significant difference) when supplied with 0.2mol CO_2_ m^–3^, and these rates all declined significantly with time of submergence (Fig. 4a, b and Table 1). On the last day of submergence, underwater P_N_ by FR13A was 3.3-fold higher than in IR42 (Fig. 4a). This higher rate in FR13A was again not evident in the *SUB1* introgression line in Swarna background (Fig. 4b; i.e. Swarna-Sub1). Although underwater P_N_ in FR13A was significantly higher than in the three other genotypes, even in FR13A towards the end of the submergence treatment the rate had declined to 40% of the initial rate (the other three genotypes had 11–19% of their initial rates). There was a positive relationship between leaf chlorophyll concentration and underwater P_N_ for three of the genotypes, but less so for Swarna (Fig. 5). As in the CO_2_ saturated condition, leaf chlorophyll concentration was positively correlated with underwater P_N_, but closer examination of the dynamics in the changes in chlorophyll as compared with changes in underwater P_N_ indicate there must also be an additional factor(s); here we assessed the potential influence of leaf gas films.

**Fig. 6.**
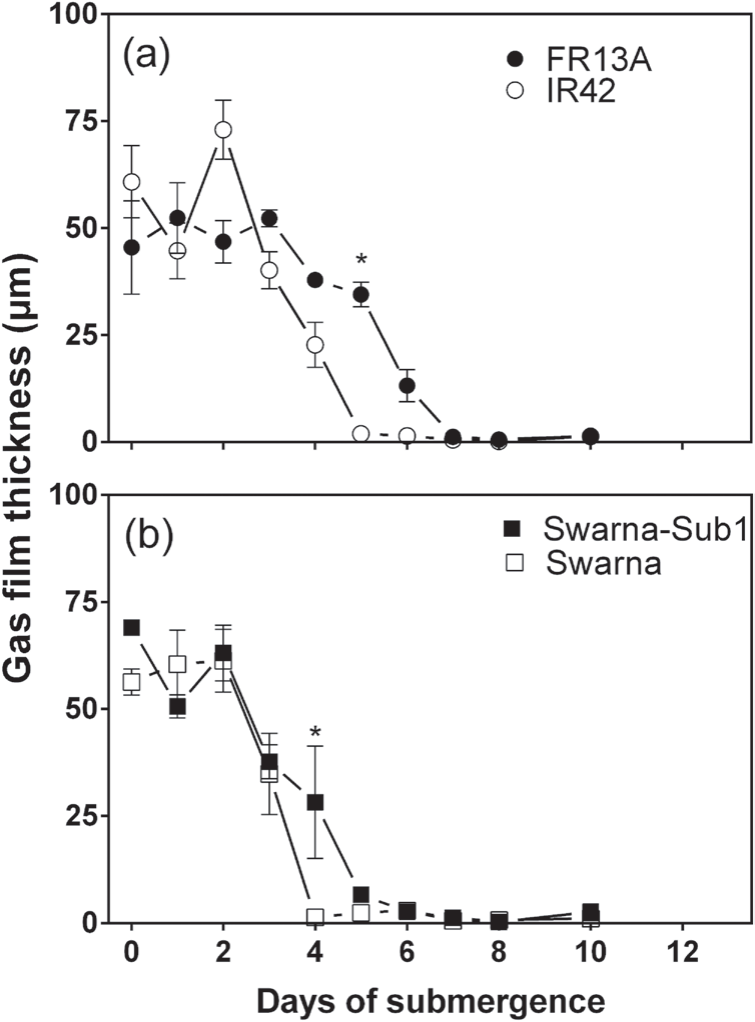
Leaf gas film thickness of four genotypes of 5–7 weeks old rice (*Oryza sativa*) with time of submergence. (a) FR13A (submergence tolerant and donor of *SUB1*) and IR42 (submergence intolerant) and (b) Swarna (submergence intolerant) and Swarna-Sub1 (submergence tolerant with *SUB1* QTL introgressed). Gas film volume was measured by determining tissue buoyancy before and after gas film removal using the method of Raskin (1983) and then divided by two-sided leaf area to obtain mean thickness (mean±SE, *n* = 4). Gas film thickness decreased significantly with time of submergence (Table 1); asterisk denotes significant differences between the two genotypes in each panel (Bonferroni test).

**Fig. 7.**
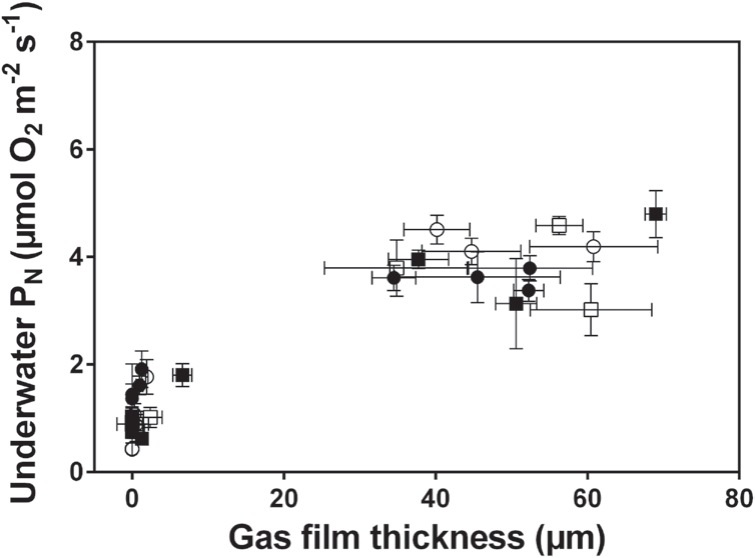
Leaf gas film thickness *versus* underwater net photosynthesis (P_N_) measured at 0.2mol CO_2_ m^–3^ of four genotypes of 5–7 weeks old rice (*Oryza sativa*). Genotypes were: FR13A (submergence tolerant and donor of *SUB1*; solid circle), IR42 (submergence intolerant; open circle), Swarna (submergence intolerant; open square), and Swarna-Sub1 (submergence tolerant with *SUB1* QTL introgressed; solid square). Spearman rank correlation analyses (one-tailed) of gas film thickness *versus* underwater P_N_ showed no significant correlations of neither all genotypes pooled nor for each individual genotype when excluding gas film thicknesses below 2 µm (the detection limit of the present method of gas film quantification). Means±SE, *n*=5.

All four genotypes initially possessed gas films on both leaf sides when submerged. These gas films were maintained near the initial thickness for the first 4 days in FR13A and IR42, and then declined with time of submergence (Fig. 6a, Table 1). The decline, however, was initially faster for IR42 than FR13A, so that gas films were lost by the 5th day in IR42 and by the 7th in FR13A. The dynamics in the reductions in thickness of the gas films were, with exception of day 4, essentially the same for Swarna-Sub1 and Swarna (Fig. 6b, “genotype × time” interactions listed in Table 1); these declines resembled those of IR42. Fig. 7 evaluates the relationship between leaf gas films thickness and underwater P_N_ using the data up to day 7 by which time gas films had been lost for all genotypes but leaf chlorophyll had not yet significantly declined; this ensures that the effect of gas films is not confounded at this stage by changes in chlorophyll concentrations. This analysis shows that the initial declines in leaf gas film thickness hardly influenced underwater P_N_ whereas underwater P_N_ was markedly lower when gas films were no longer present (Fig. 7).

### Growth, leaf sugars/starch, and survival

The present study was in a field with simulated flash-flooding causing complete submergence of 13 days. In addition to our focus here to fill the knowledge gap on underwater P_N_ and gas film persistence for these contrasting genotypes, growth during submergence, leaf sugars/starch, and survival were also evaluated. As earlier work has focused on these aspects (Mackill *et al.*, 2012), here we relegate those data to the supplementary materials (Supplementary Figs S2 and S3 available at *JXB* online).

## Discussion

FR13A has high tolerance of submergence (Singh *et al.*, 2001) and a large proportion of this tolerance is associated with the *SUB1* QTL (Mackill *et al.*, 2012). The *SUB1* QTL confers submergence tolerance in rice, assessed as survival and recovery of growth and/or yield following transient complete submergence (Jagadish *et al.*, 2012). This tolerance is associated with less elongation during submergence, higher soluble carbohydrates in shoots, and less oxidative damage post-submergence (Fukao *et al.*, 2009; Xu and Mackill, 1996; Xu *et al.*, 2006). These traits are well studied in FR13A and *SUB1* genotypes, whereas the known ability of FR13A to retain chlorophyll when submerged (Ella *et al.*, 2003b) and its influence on underwater P_N_ had not previously been evaluated. The present study shows that when submerged, FR13A retains its capacity for underwater P_N_ (CO_2_ saturated rate), whereas this capacity declined markedly in sensitive genotypes (IR42 and Swarna, Fig. 1). Nevertheless, at near ambient CO_2_ levels in floodwater, underwater P_N_ had declined in all genotypes during the second week of submergence, as leaf gas films only persisted for the first several days (Fig. 6). Regarding the *SUB1* QTL, Swarna-Sub1 also showed improved chlorophyll retention, but its capacity for underwater P_N_ was not improved, indicating that other components of the photosynthetic machinery must have been compromised. The changes in gas film presence and leaf chlorophyll concentration (and presumably other components of the photosynthetic machinery) with duration of submergence both contribute to the decline in rates of underwater P_N_ of submerged rice.

The impressive maintenance by FR13A of capacity for underwater P_N_ (CO_2_ saturated rate) during 13 days of submergence adds to the list of known traits associated with submergence tolerance in this genotype, being much higher than in Sub1 introgression lines (Neeraja *et al.*, 2007; Singh *et al.*, 2009). FR13A is known to possess four more, but minor, QTLs associated with submergence tolerance (Nandi *et al.*, 1997). Submerged rice can suffer leaf chlorosis, a condition triggered by ethylene accumulation, but chlorosis is less in tolerant (e.g. FR13A) as compared with sensitive (e.g. IR42) genotypes (Ella *et al.*, 2003b; Jackson *et al.*, 1987). The present underwater P_N_ measurements add functional data to extend the previous observation of better chlorophyll retention in FR13A as compared with IR42 (Ella *et al.*, 2003b). An earlier study had indicated a significant decline in photosynthetic capacity already after 1 day (IR42) and 3 days (FR13A) of submergence (Smith *et al.*, 1988), but this earlier work used an IRGA to measure leaves soon after return to air. By contrast, the present study measured photosynthesis under water (Pedersen *et al.*, 2013) and IR42 declined in photosynthetic capacity (i.e. CO_2_ saturated rate) only during the second week of submergence (Fig. 1). The fast declines in photosynthetic rates observed by Smith *et al.* (1988) were not associated with changes in leaf chlorophyll, whereas in our longer term study there were strong positive correlations between reductions in leaf chlorophyll concentrations and reduced capacity for underwater P_N_ (Fig. 3).

Underwater P_N_ at 0.2mol CO_2_ m^–3^ (representative of ambient in submergence situations) was not, however, preserved as well as underwater P_N_ at high CO_2_ (5mol CO_2_ m^–3^) for the leaves of submerged rice. The declines with time in underwater P_N_ of the various genotypes at 0.2mol CO_2_ m^–3^ were probably due to the loss of leaf gas films after 4–6 days of submergence; loss of gas films would decrease the uptake of CO_2_ from the floodwater (c.f. Pedersen *et al.*, 2009). Gas films persisted on the submerged leaves for 4–6 days depending on genotype and the loss of leaf gas films were strongly linked to a steep decline in underwater P_N_ at 0.2mol CO_2_ m^–3^ for all four genotypes (Figs 4 and 6). By contrast, lamina chlorophyll concentration did not significantly decrease until after the leaf gas films had disappeared and so the substantial declines in underwater P_N_ during the initial 5 days of submergence were therefore unlikely to have been caused by chlorophyll degradation. Leaf gas films increase underwater gas exchange and thus CO_2_ entry to sustain rates of underwater P_N_ (Colmer and Pedersen, 2008b; Pedersen *et al.*, 2009; Winkel *et al.*, 2011). Moreover, modelling of O_2_ entry during darkness into respiring rice leaves with or without gas films has further demonstrated that the resistance to O_2_ exchange with the floodwater is reduced by the presence of gas films, with assessments also of the various resistance components in the pathway(s) (Verboven *et al.*, 2014).

Leaf gas films have been shown to enhance internal aeration of belowground tissues during complete submergence (Pedersen *et al.*, 2009; Winkel *et al.*, 2013; Winkel *et al.*, 2011). It was recently shown that even low rates of underwater P_N_ greatly influence root O_2_ status during daytime for Swarna-Sub1 during 2 days of submergence in a field (Winkel *et al.*, 2013). Thus, retention and persistence of leaf gas films by submerged plants is likely to be beneficial, but factors involved in the degradation of leaf gas films during prolonged submergence require additional study. Leaf gas films might also be an effective barrier against infections and we speculate when lost this will facilitate contact and colonization by microorganisms in the floodwater. It can be hypothesized that once the leaf gas films have been lost the process of tissue deterioration speeds up, eventually leading to tissue death. Superhydrophobic leaf surfaces are hypothesized to be an adaptation for leaves to self-clean and facilitate water to roll off leaves in air when it rains to prevent covering of leaves by a film of water (Neinhuis and Barthlott, 1997), as a water layer on a leaf surface would reduce gas exchange and thus photosynthesis, and also enhance the likelihood of bacteria and fungi infecting leaves (Koch *et al.*, 2009). The leaf gas film persistence was moderately longer in FR13A and our data show that underwater P_N_ at a near ambient CO_2_ concentration was strongly enhanced by leaf gas film presence. Thus, we wonder if there is larger diversity of gas film retention and persistence in lowland rice than documented in the present study.

Pedersen *et al.* (2009) demonstrated the essential role of leaf gas films on sugar status of completely submerged rice and Winkel *et al.* (2013) showed the importance of underwater P_N_ for internal aeration in roots of submerged rice. The mechanisms determining longevity of leaf gas films should be further elucidated and rice germplasm screened for longer leaf gas film persistence during submergence, as this trait could potentially increase carbohydrate status and internal aeration owing to increased underwater P_N_ during prolonged submergence. Furthermore, studies are needed to investigate the extent of gas films persistence as related to various weather and floodwater characteristics that affects survival in the field e.g. conditions as noted in Das *et al.* (2009) and in Colmer *et al.* (2014).

## Supplementary data

Supplementary data are available at *JXB* online

Figure S1. Leaf lamina porosity

Figure S2. Leaf lamina sugars and starch

Figure S3. Relative growth rate and survival

Supplementary Data
