# Supplementary material for: Gas film retention and underwater photosynthesis during field submergence of four contrasting rice genotypes
Source: J Exp Bot. 2014 Apr 23;65(12):3225–33. doi: 10.1093/jxb/eru166 (PMC4071835; doi:10.1093/jxb/eru166)
Supplement: Supplementary Data [file supp_65_12_3225__index.html]

Gas film retention and underwater photosynthesis during field submergence of four contrasting rice genotypes — Gas film retention and underwater photosynthesis during field submergence of four contrasting rice genotypes — Supplementary Data 

# Gas film retention and underwater photosynthesis during field submergence of four contrasting rice genotypes

## Supplementary Data

Data files

**Files in this Data Supplement:**

- Supplementary Data - Supplementary Data
