# Supplementary material for: Gas film retention and underwater photosynthesis during field submergence of four contrasting rice genotypes
Source: J Exp Bot. 2014 Apr 23;65(12):3225–33. doi: 10.1093/jxb/eru166 (PMC4071835; doi:10.1093/jxb/eru166)
Supplement: Supplementary Data [file supp_eru166_jexbot123745_file001.pdf]

Figure 1S

Leaf lamina porosity of four genotypes of 5-7 week old rice (*Oryza sativa*) during 13 d of submergence in a field pond at IRRI, the Philippines. Leaf lamina porosity decreased significantly with time of submergence (two-way ANOVA, P < 00001) and also differed between genotypes (two-way ANOVA P = 0.0002). * denotes significant differences between the two genotypes in each panel (Bonferroni).

Methods

After the first measurement (the buoyancy with gas film intact) segments were brushed with a dilute solution of Triton X-100 (0.01% v/v of Triton X-100 in artificial floodwater, composition given above) to eliminate hydrophobicity so that gas films were removed cf (Pedersen et al., 2009b) and then re-measured, after which vacuum infiltration of the gas spaces with water performed and again re-measured, using the method of Raskin (1983) with equations as modified by Thomson *et al.* (1990).

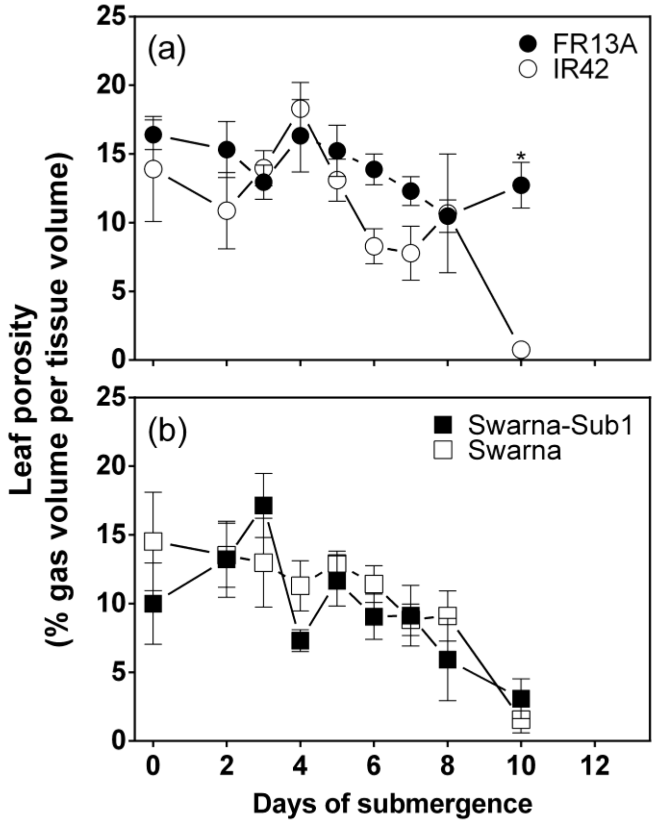

Figure 2S

Leaf lamina sugar (ab) and starch concentrations (cd) of four genotypes of 5-7 week old rice (*Oryza sativa*) during 13 d of submergence in a field pond at IRRI, the Philippines. Leaf lamina sugar and starch concentration decreased significantly with time of submergence and were significantly different between genotypes (two-way ANOVA $P < 0.0001$; applies to all panels and genotypes). * denotes significant differences between the two genotypes in each panel (Bonferroni).

Methods

Tissue sugar and starch concentrations were measured on pooled leaf lamina samples (i.e. all the lamina of one individual plant constituted one sample, sheaths were excluded) from five plants of each genotype (n = 5). The tissue samples were flash frozen in liquid N_2, freeze dried for 48 h, cut then ground to a fine powder and then stored at room temperature inside a desiccator until analysis. Sugars were extracted from tissue samples boiled twice in 80% ethanol with reflux for 20 min. Total sugar levels in the extracts were measured using anthrone (Fales, 1951) and a spectrophotometer (UV-VIS 1800, Shimadzu, Kyoto, Japan). The residue was used for starch analysis, in brief washed 3 to 5 times, solubilised in boiling water for 3 h with further hydrolysis catalyzed by amyloglucosidase (Sigma Chemicals, St. Louis, MO, USA). The hydrolyzed starch was subsequently analyzed for free sugars using Sigma Chemicals peroxidase-glucose oxidase enzyme-colour reagent with o-dianisidine dihydrochloride (Setter *et al.*, 1994).

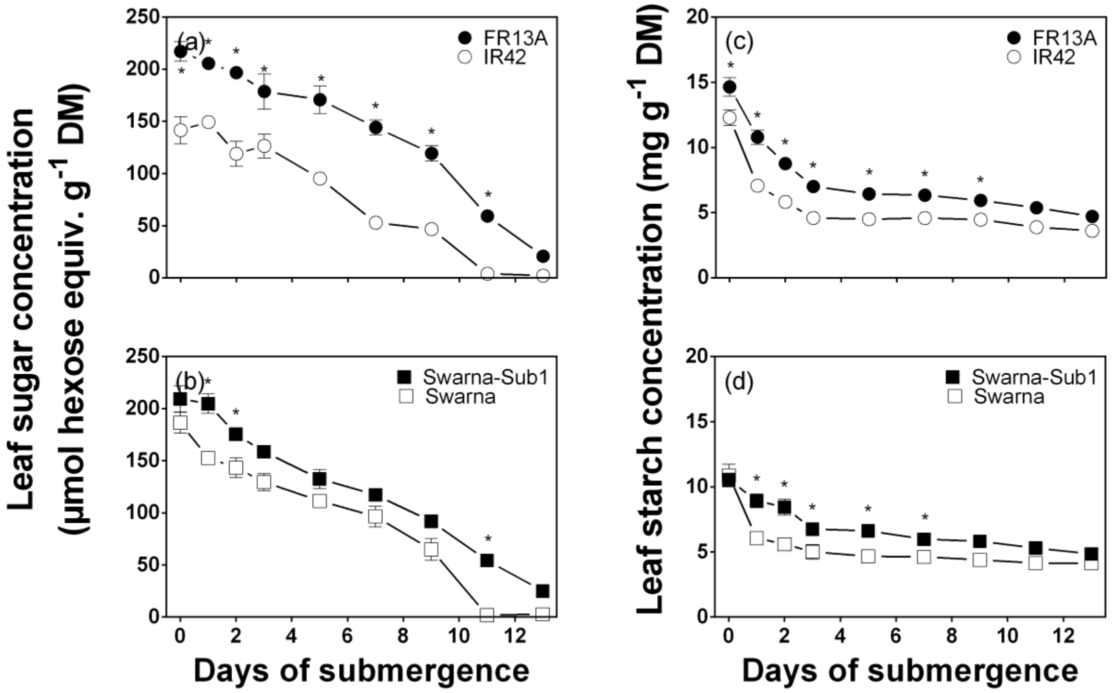

Figure 3S
Relative shoot growth rate (RGR) and survival of four rice genotypes (*Oryza sativa*) during 13 d of submergence in a field pond at IRRI, the Philippines. RGR (a) was calculated between initial values and after 7 d of submergence and between 7 and 13 d of submergence (mean ± SE, n= 5). Survival (a) was scored after 16 d of recovery following de-submergence on initial populations minus the plants that were harvested for samples (1-way ANOVA, P = 0.0143, followed by Tukey test with letters indicating significant difference between genotypes, n = 842-942).

Methods
Shoot dry mass (DM) was measured on harvested plants of all 4 genotypes (n = 5 for each genotype). DM was measured by flash freezing the samples in liquid N_2 and then freeze drying for 48 h and storing at -80 °C in bags with desiccant until samples were weighed.

Scoring of survival was done by the visual appearance of the plants, when a plant had at least 1 new leaf after de-submergence it was assessed as surviving. There were between 842 and 942 plants of each genotype at the beginning of the submergence treatment. Survival was calculated as a percentage of live plants compared to the initial number of plants.

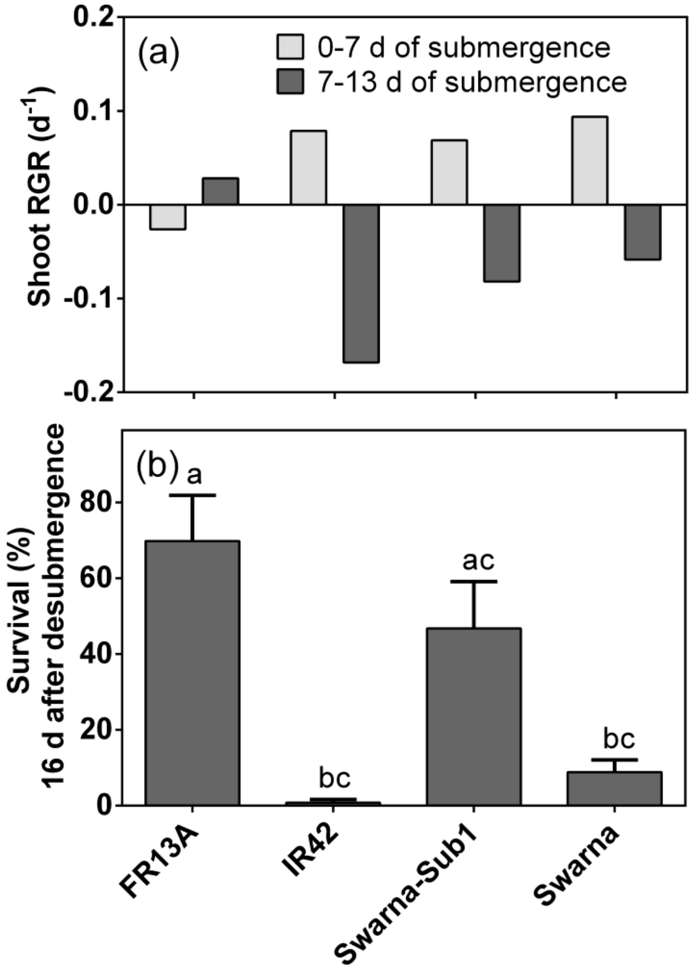

References

Fales FW. 1951. The assimilation and degradation of carbohydrates by yeast cells. *Journal of Biological Chemistry* **193**, 113-124.

Raskin I. 1983. A method for measuring leaf density, thickness and internal gas. *Hortscience* **18**, 698-699.

Setter T, Ella E, Valdez A. 1994. Relationship between coleoptile elongation and alcoholic fermentation in rice exposed to anoxia. II. Cultivar differences. *Annals of Botany* **74**, 273-279.

Thomson CJ, Armstrong W, Waters I, Greenway H. 1990. Aerenchyma formation and associated oxygen movement in seminal and nodal roots of wheat. *Plant, Cell & Environment* **13**, 395-403.
